# Supplementary material for: Real-space imaging of confined magnetic skyrmion tubes
Source: Nat Commun. 2020 Apr 7;11:1726. doi: 10.1038/s41467-020-15474-8 (PMC7138844; doi:10.1038/s41467-020-15474-8)
Supplement: Supplementary file 1 — Supplementary Information [file 41467_2020_15474_MOESM1_ESM.pdf]

Real-space imaging of confined magnetic skyrmion tubes

Birch et al.

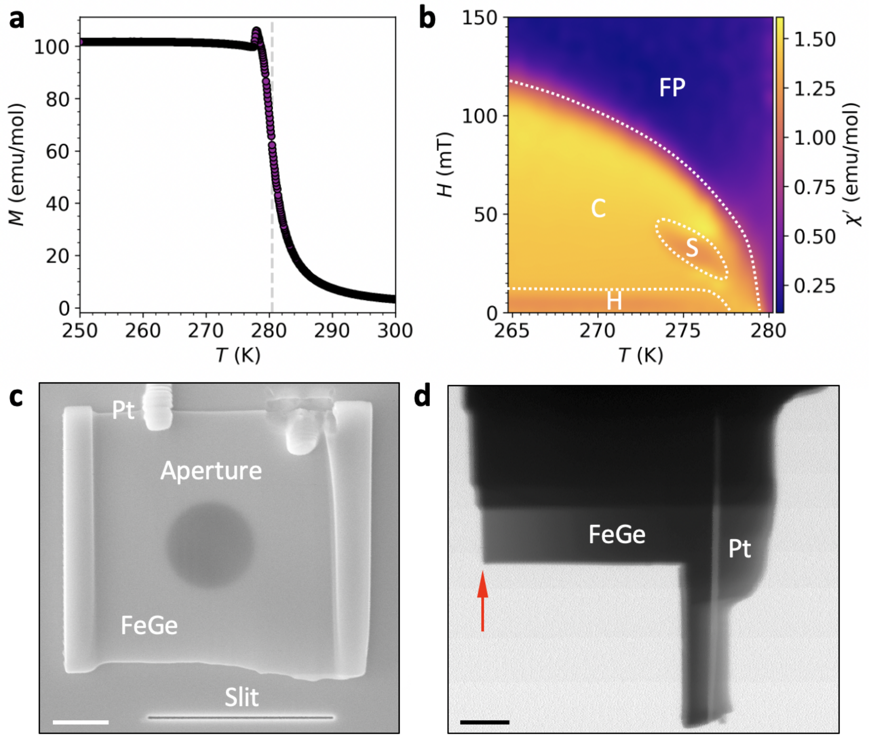

Supplementary Figure 1 | **Bulk and thin lamella FeGe samples.** **a**, A plot of magnetisation against temperature as measured for the bulk FeGe single crystal. The grey dotted line marks the determined T_C of ~ 280.5 K. **b**, The magnetic phase diagram of the bulk FeGe sample, as determined by AC susceptibility measurements. The extent of the helical (H), conical (C), skyrmion lattice (S) and field polarised (FP) states are labelled. **c**, Scanning electron micrograph of sample 1. **d**, Scanning transmission x-ray micrograph of sample 2, in the same orientation as in Fig. 4**a** of the main text . The red arrow indicates the location of the observed magnetic skyrmion tubes. Scale bars, 2 μm.

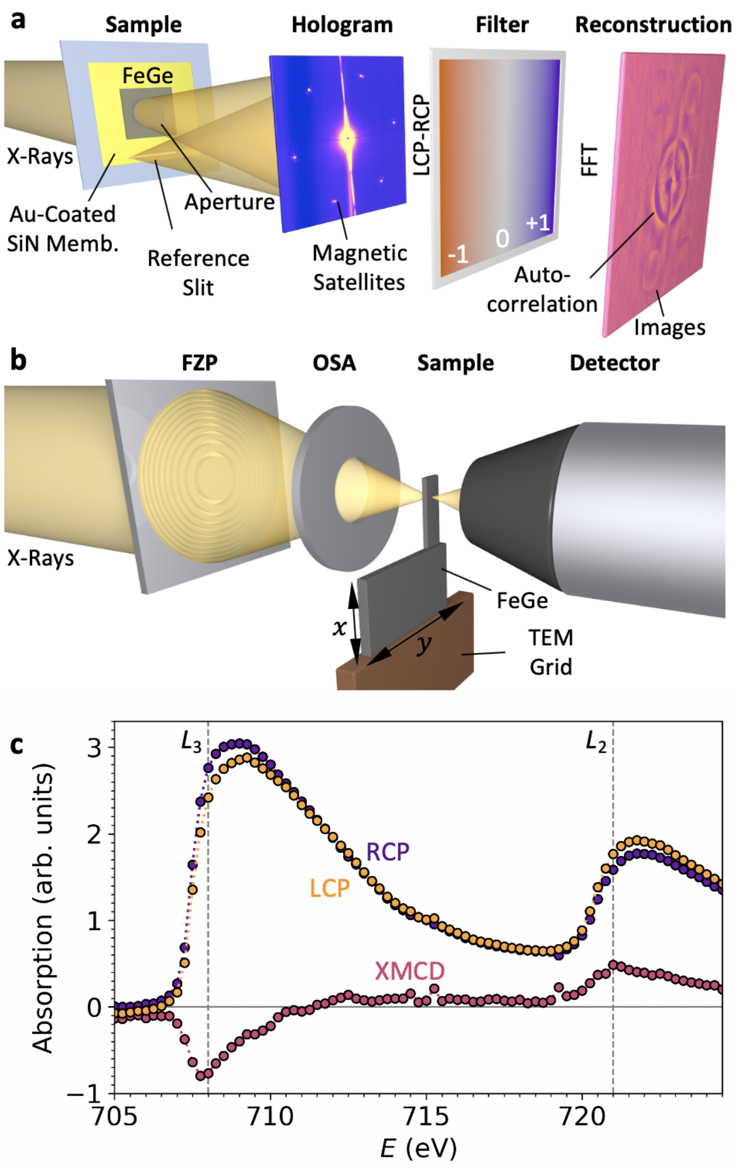

Supplementary Figure 2 | **Experimental setup diagrams and x-ray spectra.** **a**,**b**, Schematic illustrations of the magnetic x-ray diffraction and holographic imaging experiments (**a**), and the scanning transmission microscopy setup (**b**). **c**, Absorption spectrum of the Fe L_3 and L_2 edges, measured for both left (LCP) and right (RCP) circularly polarised x-rays. The difference is the XMCD spectrum, also displayed multiplied by two.

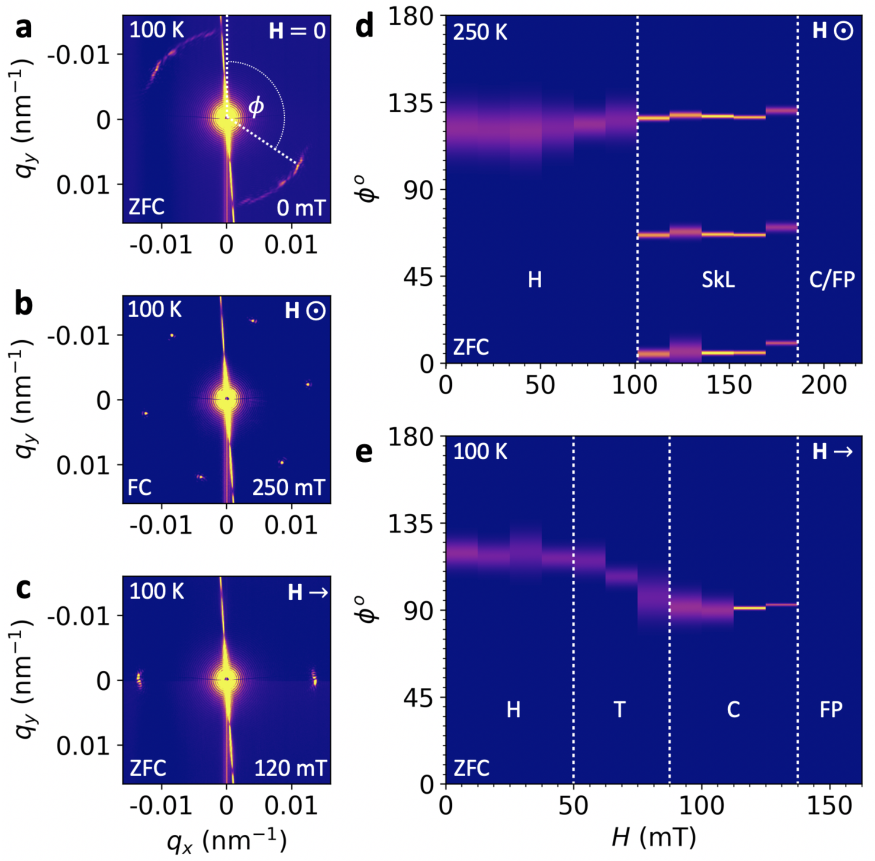

Supplementary Figure 3 | **Magnetic phase diagram determination. a**-**c**, CCD images of the magnetic x-ray diffraction patterns obtained for the helical (H), skyrmion lattice (SkL) and conical (C) states respectively. The central yellow spot is the main x-ray beam, blocked by a beamstop, while the smaller outer peaks are the magnetic diffraction satellites. **d**,**e**, Fitted angular profiles of the magnetic diffraction patterns plotted as a function of applied out-of-plane and in-plane magnetic field respectively. Determined boundaries between the magnetic states are labelled and shown by the dashed white lines.

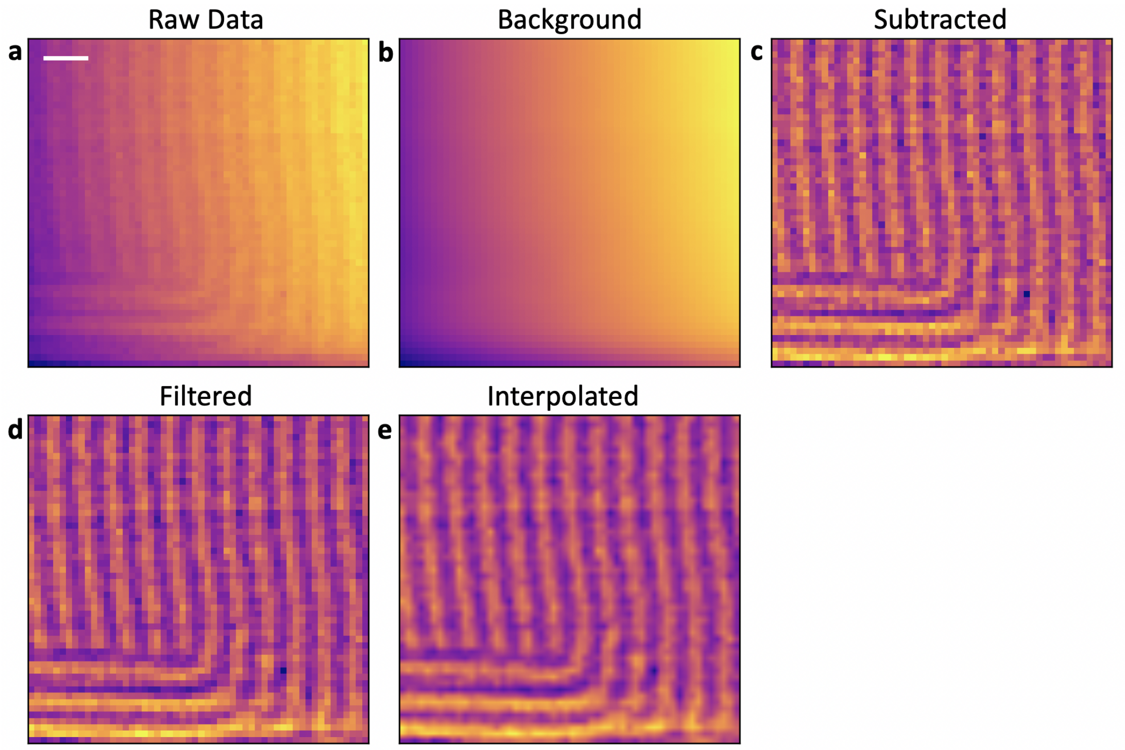

Supplementary Figure 4 | **Processing performed for image presentation.** **a**, The raw STXM data recorded with a single x-ray polarisation. **b**, The background image produced by performing a wide Gaussian filter to the raw data. **c**, The image formed by subtracting the background image from the raw data. **d**, The image formed after applying a Gaussian filter to the subtracted data. **e**, The image after applying a hamming interpolation. Scale bar, 100 nm.

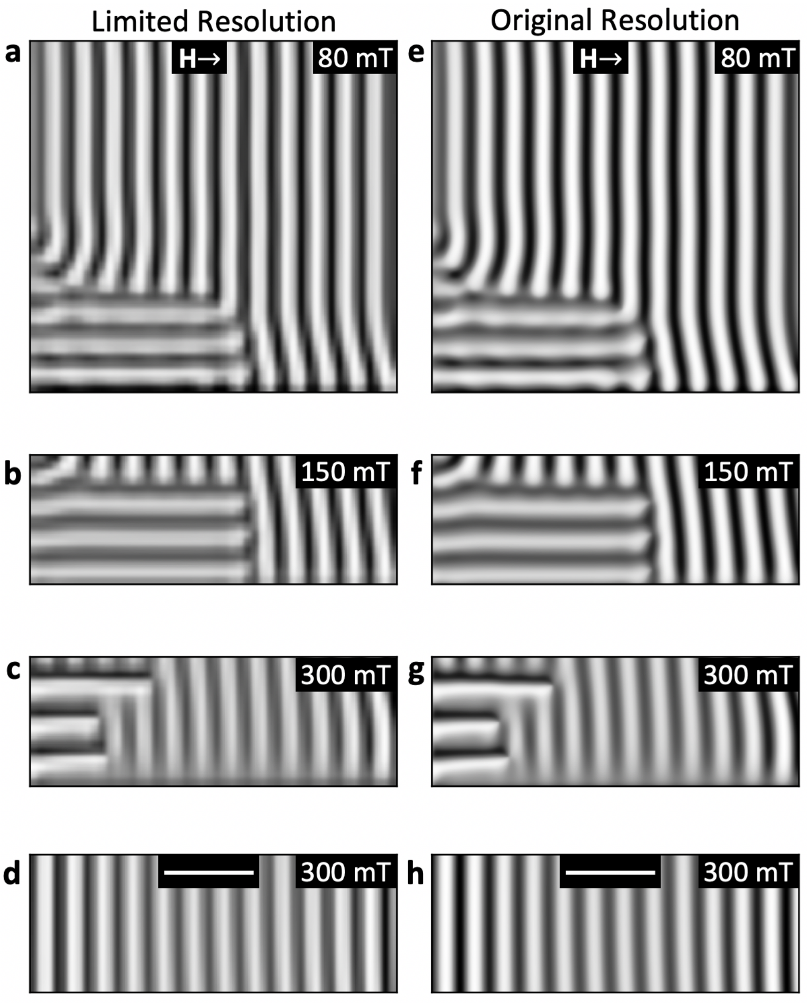

Supplementary Figure 5 | **Resolution of simulated images. a-d**, Resolution-limited simulated images of the skyrmion tube state, with a 20 nm pixel size. **e-h**, Original resolution of the simulated skyrmion tube images, with a pixel size of 4 nm. Scale bars, 200 nm.

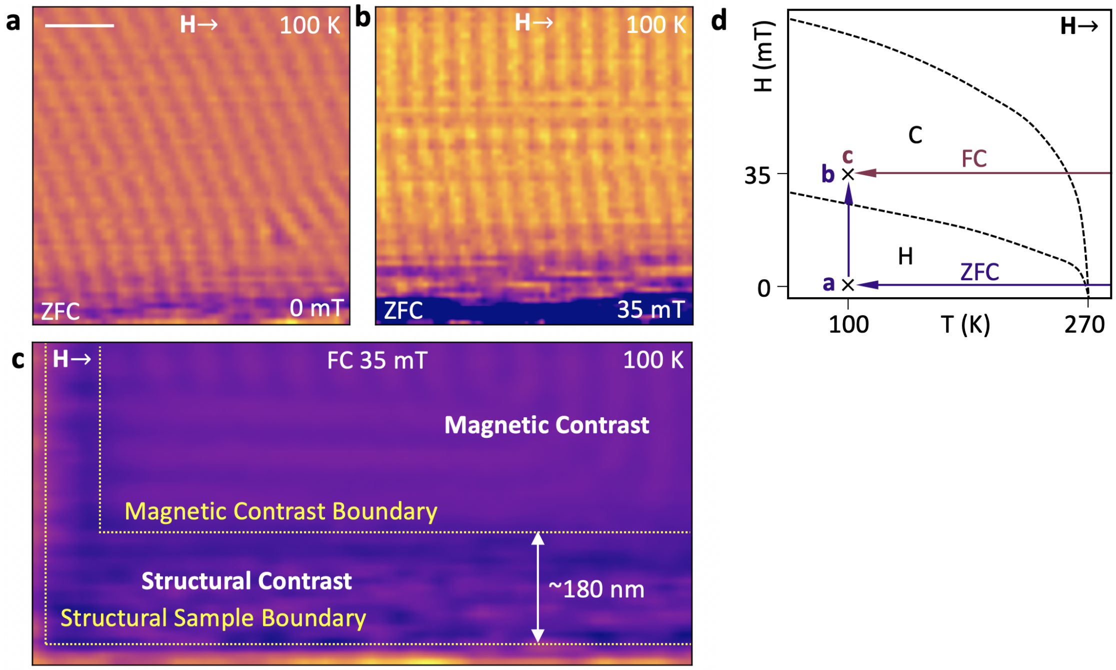

Supplementary Figure 6 | **Location of the magnetic skyrmion tubes. a-c**, Scanning transmission x-ray microscopy images of the same corner area of the sample recorded after ZFC (**a**,**b**) and FC (**c**) procedures. The limit of the magnetic contrast region and the structural edge of the sample are depicted by yellow dashed lines in **c**. **d**, Illustrative phase diagram depicting the field-temperature paths taken for each measurement. Scale bar, 200 nm.

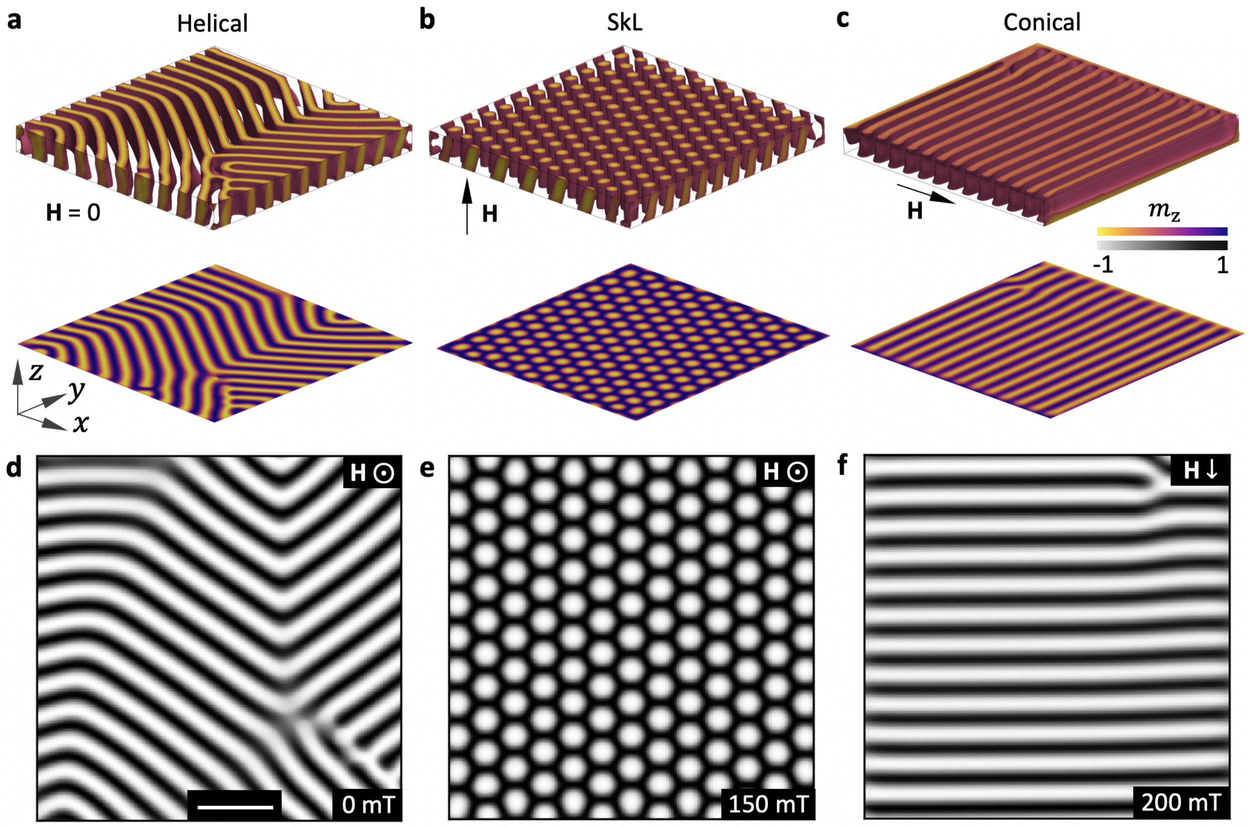

Supplementary Figure 7 | **Micromagnetic simulations of chiral spin textures. a-c**, Three dimensional visualisations of the helical, SkL and conical states presented in Fig. 2 in the main text, created by plotting cells with m_z components between -1 and 0. **e-f**, Simulated images of each simulated state, as shown in Fig. 2 of the main text. Scale bar, 200 nm.

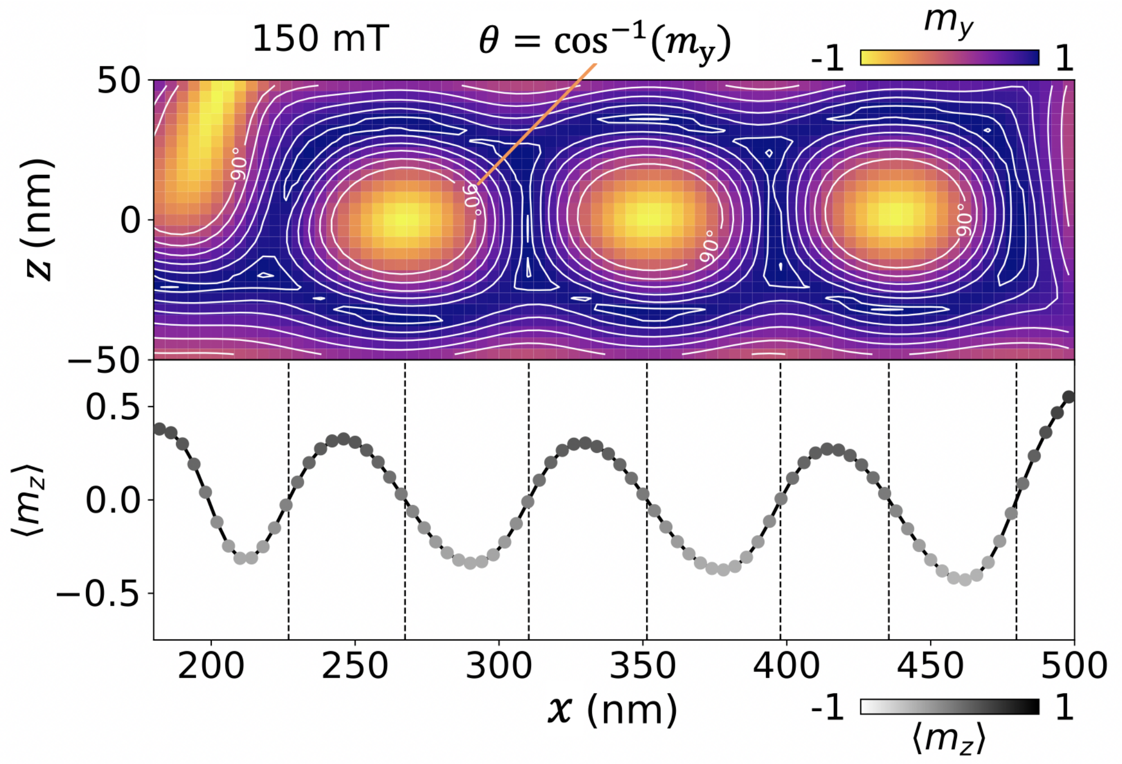

Supplementary Figure 8 | **Calculating the simulated images.** The m_y component of a cross section through the simulated skyrmion tube state at 150 mT is plotted in the top panel. Isocontours highlight the lines of constant m_{y}. The calculated average out-of-plane component, $\langle m_{\mathrm{z}} \rangle$, is plotted in the bottom panel. Vertical dotted lines indicate positions of maximum/minimum m_{y}.

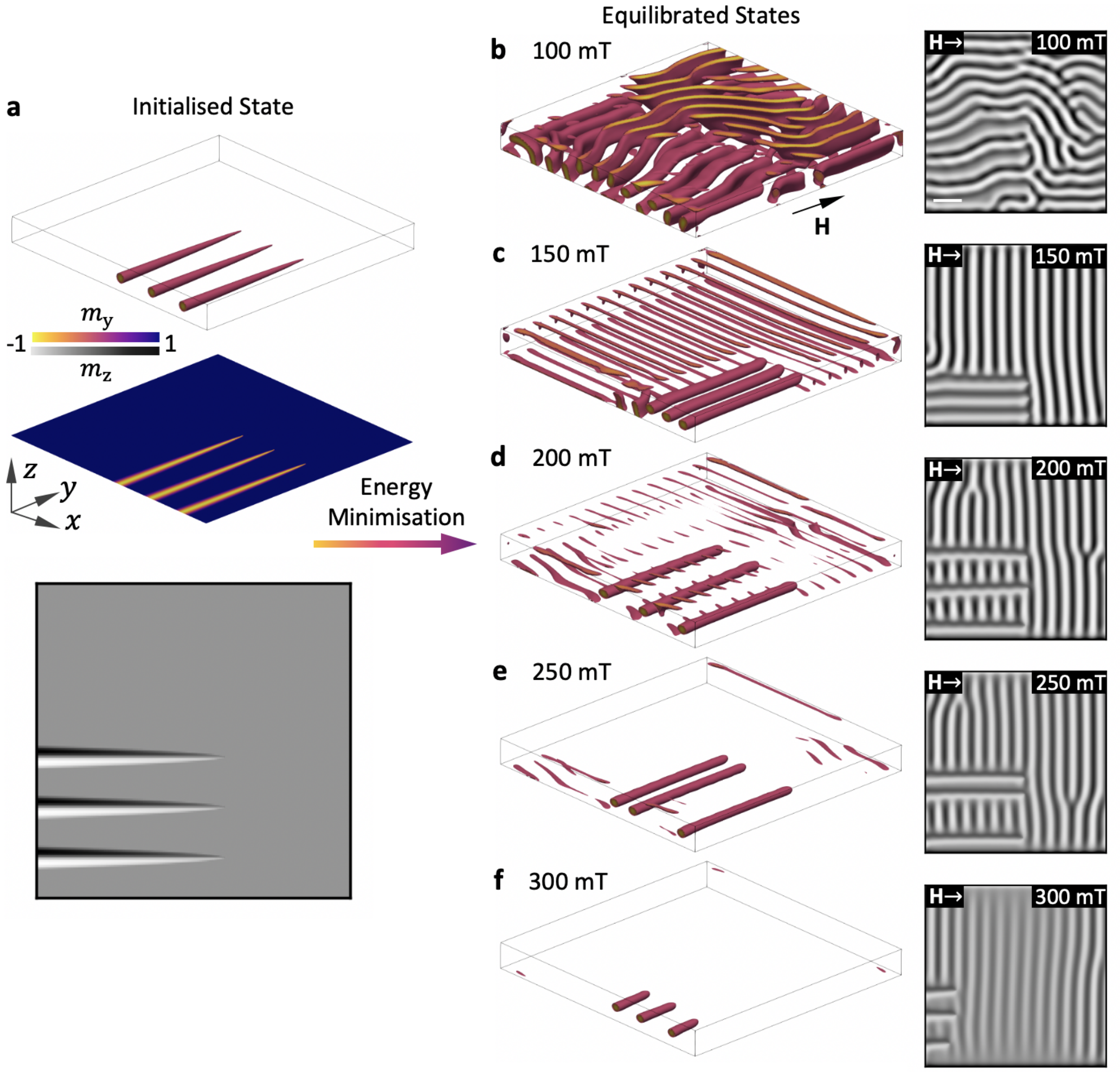

Supplementary Figure 9 | **Initialisation of skyrmion tube state.** **a**, Visualisation of the initial skyrmion tube state, featuring three paraboloid-shaped skyrmion tube precursors in a ferromagnetic background. The simulated x-ray image is shown below. **b**-**f**, Visualisations, created by plotting cells with m_y components between -1 and 0, and simulated x-ray images of the magnetic state after energy minimisation at a range of in-plane applied magnetic fields. Scale bar, 100 nm.

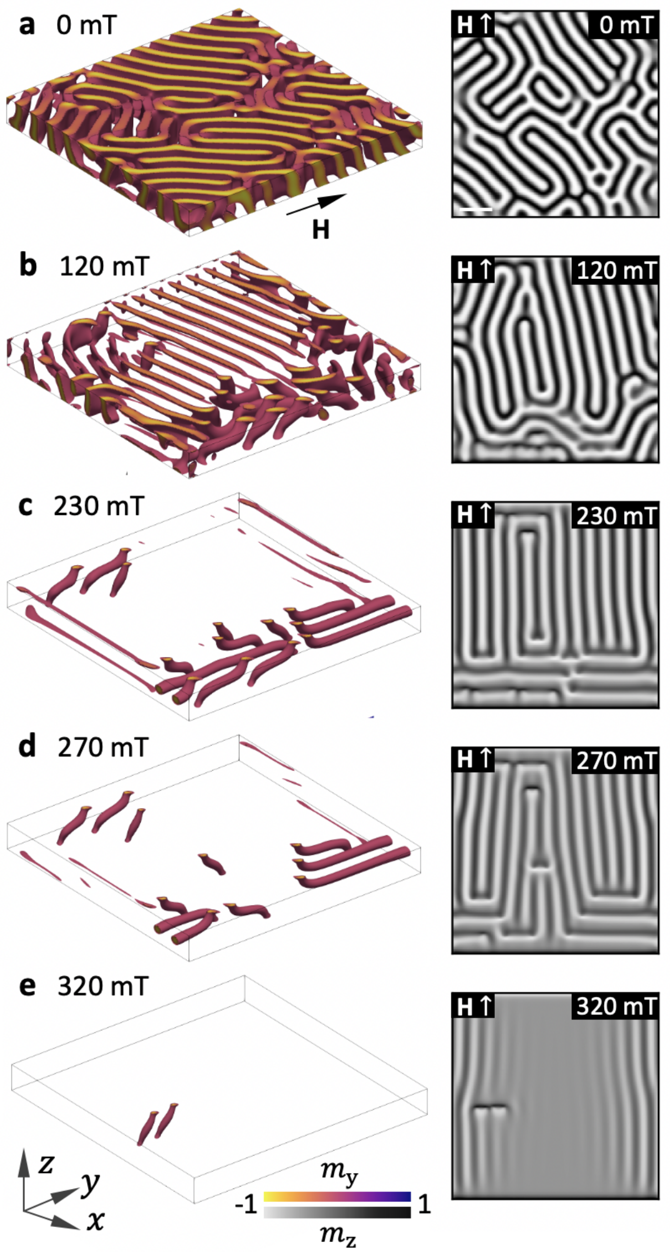

Supplementary Figure 10 | **In-plane magnetic field simulation from random initial state. a-e**, Selected three dimensional visualisations, created by plotting cells with m_y components between -1 and 0, and simulated images of the magnetic state during an in-plane field sweep from an initially randomised state. Scale bar, 100 nm.

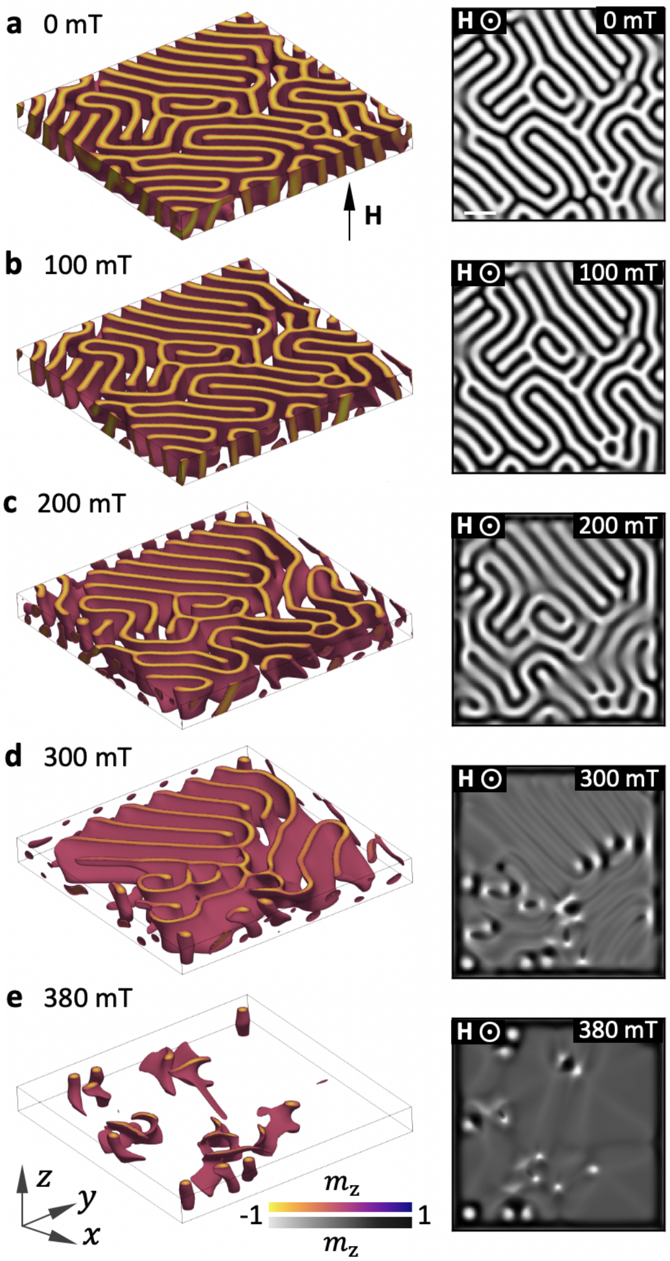

Supplementary Figure 11 | **Out-of-plane magnetic field simulation from random initial state. a-e**, Selected three dimensional visualisations, created by plotting cells with m_z components between -1 and 0, and simulated images of the magnetic state during an out-of-plane field sweep from an initially randomised state. Scale bar, 100 nm.

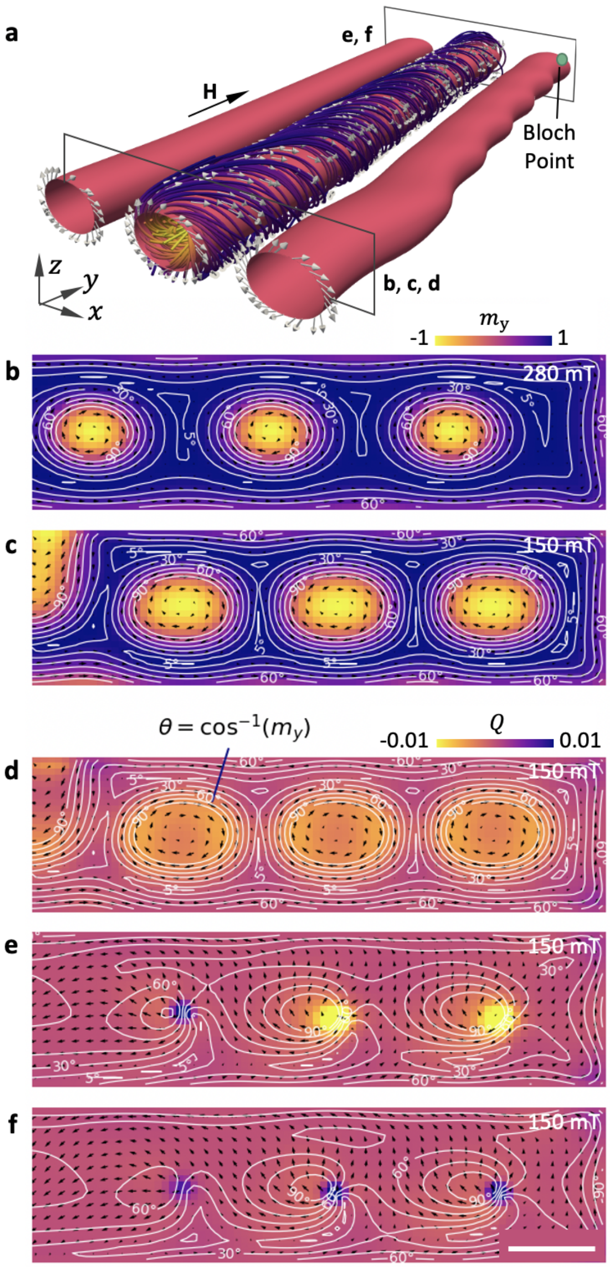

Supplementary Figure 12 | **Isocontours and topological charge of skyrmion tubes.** **a**, Three dimensional visualisation of the skyrmion tube spin texture at 150 mT. **b**,**c**, The m_{y} component of cross sections through the simulated skyrmion tube states at 280 and 150 mT. Isocontours exhibit the lines of constant m_{y}. **d**-**f**, Calculated topological charge density of cross sections through the simulated skyrmion tube state at 150 mT close to the edge of the sample (**d**), and at the position of the magnetic Bloch point (**e**,**f**). Scale bar, 50 nm.

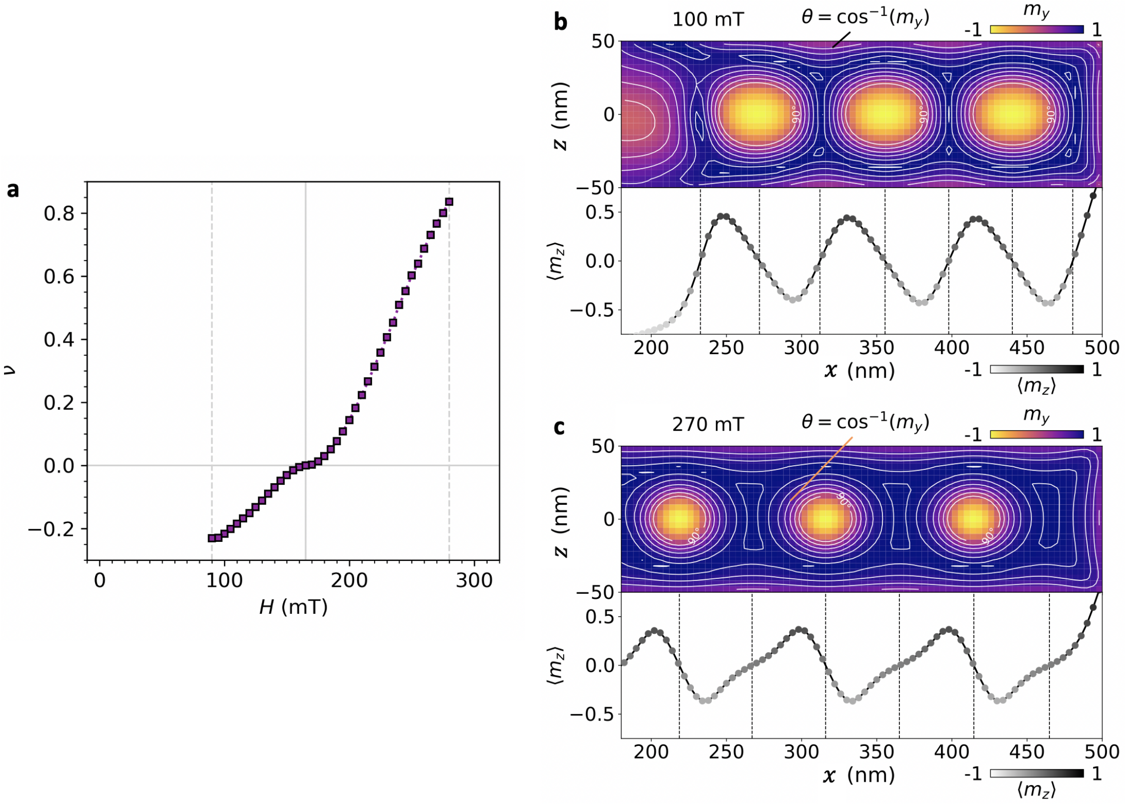

Supplementary Figure 13 | **Distortion of the skyrmion tube magnetisation profiles.** **a**, The parameterised distortion, ν, of the out-of-plane magnetisation profile of the skyrmion tube state as a function of applied magnetic field. **b**,**c**, The m_y component of cross sections through the simulated skyrmion tube states at 100 and 270 mT are plotted in the top panels. Isocontours highlight the lines of constant m_y. The calculated average out-of-plane component, $\langle m_\mathrm{z} \rangle$, is plotted in the bottom panels. Vertical dotted lines indicate positions of maximum/minimum m_y.

Supplementary Note 1: Sample preparation and characterisation

The FeGe single crystals were grown by the chemical vapour transport method with iodine as the transport agent, as described by Richardson[1]. We performed initial magnetometry measurements to characterise the bulk single crystal FeGe sample, displayed in Supplementary Fig. 1**a** and **b**. The T_{C} of the sample, defined as the point of greatest slope in M, was found to be 280.5 K, as determined by the magnetisation vs temperature data in Supplementary Fig. 1**a**. The thin lamella samples in the main text were observed to have a Curie Temperature, T_{C}, of 270 K. This difference is likely due to two factors: a temperature offset of the lamella sample from the temperature sensor during the x-ray measurements, or a reduction in T_{C} due to ion implantation damage to the lamella during the fabrication process. A magnetic phase diagram of the crystal is shown in Supplementary Fig. 1**b**, highlighting the limited extent of the SkL state in the bulk when compared to the out-of-plane field lamella phase diagram in Fig. 1**a** in the main text. The phase diagram colourmap plots the AC susceptibility measured during field sweeps after zero field-cooling at each temperature. A region with a characteristic dip in the AC signal is a well known indicator of the SkL state[2].

Microscopy images of the thin lamella samples of FeGe investigated in the main text are shown in Supplementary Fig. 1**c** and **d**. Sample 1, shown in Supplementary Fig. 1**c**, consists of a $\sim$120 nm thick lamella fabricated by focused ion beam. This lamella was placed over a 3 µm aperture in a Si_4N_3 membrane, which was sputter coated on the reverse side with $\sim$600 nm of Au, and fixed in position by a single Pt deposition weld. A 20 nm thick reference slit was milled 3.5 µm from the sample aperture. The Au layer serves to block the majority of the incoming x-

ray beam, leaving only scattering from the sample aperture and the reference slit incident on the CCD. Sample 2, shown in Supplementary Fig. 1**d**, consists of a $\sim$120 nm thick lamella again fabricated by focused ion beam. The lamella was further ion milled into the L-shape shown in the sample image. The contrast in the image demonstrates that the bottom left corner of the sample, highlighted by the red arrow, is the thinnest part of the sample. The SkT spin texture imaged in the main text was located in this corner.

Supplementary Note 2: Experimental setups

The experimental setups for the magnetic x-ray diffraction, x-ray holography[3,4] and STXM[5,6] are shown in Supplementary Fig. 2**a** and **b**, and are described in the methods section of the main text. Figure S2**c** displays the x-ray absorption spectrum measured on sample 2. The sample absorption is resonantly enhanced when the energy of the incident x-rays are tuned to the Fe L_3 and L_2 edges[7]. A difference in absorption between left and right circularly polarised x-rays indicates x-ray magnetic circular dichroism (XMCD), and this effect is exploited to achieve contrast in magnetic STXM imaging. In the STXM measurements, in order to achieve magnetic contrast at these energies without suffering from spectral compression due to the increased absorption at the resonant edge, we found that the thickness of the FeGe sample was required to be less than 150 nm. Details of the transport of intensity equation analysis used to produce the LTEM images can be found in previous work[8].

Supplementary Note 3: Determination of magnetic phase diagrams

Magnetic x-ray diffraction patterns obtained from sample 1 are shown in Supplementary Fig. 3**a**-**c**. The two broad magnetic satellites in the Supplementary Fig. 3**a** are characteristic of a helical state with weak alignment along a preferred crystalline axis. The six magnetic peaks in Supplementary Fig. 3**b** indicate the presence of the SkL state under an out-of-plane aligned field, while the two peaks aligned to the in-plane applied field in Supplementary Fig. 3**c** are representative of the conical state. By recording the CCD images as a function of applied magnetic field after ZFC, such diffraction patterns were used to plot the phase diagram in Fig. 1**a** and **b** in the main text. For each diffraction pattern, the total scattering intensity at each azimuthal angle ϕ, as shown in Supplementary Fig. 3**a**, was summed over a q range from 0.01 to 0.015 nm^{-1} and fitted with a Gaussian distribution. Figure S3**d** displays the fitted intensity and angular position of one of each pair of magnetic peaks as a function of applied out-of-plane magnetic field at 250 K. The broad magnetic peak of the helical state transforms to three narrow magnetic peaks in the SkL state, indicating a transition from multiple helical domains with differing orientations to a highly ordered, single SkL domain state. A similar data set for an in-plane magnetic field at 100 K is shown in Fig S3**e**, illustrating the transition of the broad helical magnetic peak through a rotation of the spin texture, to the conical state.

Supplementary Note 4: Image presentation

Due to the slight variation in sample thickness across the second lamella sample, the intensity of transmitted x-rays varied across each STXM image, as shown by the example raw image in Supplementary Fig. 4**a**. We therefore performed a background smoothing process in order to allow the magnetic contrast in the images to be displayed clearly, as in the main text. In order to achieve this, we applied a Gaussian filter with a width of 3 pixels to each STXM image, producing a blurred image which effectively acts as a background mapping the transmitted x-ray intensity variation due to sample thickness, as shown in Supplementary Fig. 4**b**. By subtracting this background image from the original image, an image with uniform background contrast is acquired, revealing the magnetic contrast as seen in Supplementary Fig. 4**c**. We then applied a Gaussian filter with a width of 0.5 pixels and some minor interpolation, producing the final image in Supplementary Fig. 4**d**. In principle, all structural contrast could be eliminated by subtracting a true background image, collected under the same imaging conditions but at a temperature above T_{C}. Alternatively, a subtraction of magnetic contrast images measured with opposite x-ray polarisation would leave only magnetic contrast. In practice, we found alignment of the corresponding images challenging due to sample and beam drift, as well as the large size of the imaging pixels relative to the magnetic structures.

The simulations were performed with a discretisation of 4 nm. To facilitate realistic comparison of these simulated images to the experimental data, we limited their resolution to 20 nm. We achieved this by averaging each 5 $\times$ 5 array of the 4 nm pixels in the simulated image into a 20

nm pixel. In Supplementary Fig. 5, the simulated images with limited resolution presented in the main text are shown side by side with images at their original resolution.

Supplementary Note 5: Location of magnetic skyrmion tubes

Further STXM images are displayed in Supplementary Fig. 6. Comparative STXM images of the corner region of the second FeGe lamella sample are shown after ZFC, revealing the local orientation of the helical state in Supplementary Fig. 6**a**, and after the application of an in-plane field of 35 mT, showing formation of the conical state along the magnetic field direction in Supplementary Fig. 6**c**. In comparison, The position of the observed SkT spin texture after FC from 270 K to 100 K at 35 mT is exhibited in Supplementary Fig. 6**a**. A schematic illustration of temperature-field path taken during these measurements is shown in Supplementary Fig. 6**d**. A region which exhibited no magnetic contrast before the structural edge of the sample is highlighted by the dashed yellow lines. This edge region can be composed of redeposited or damaged FeGe which builds up at the edge of the sample during ion milling, leading to observed structural contrast between the limit of the ordered magnetic contrast and the structural edge of the sample.

Supplementary Note 6: Micromagnetic simulations

To generate the simulated states shown in Fig. 3 of the main text, a precursor state was initiated and then relaxed using an energy minimisation algorithm at varied applied field strengths. To initialise the SkL state a triple-q model was used. In the case of the conical and helical states, the simulation was initialised with one-dimensional spirals with a periodicity 70 nm. The top three

dimensional visualisations in Supplementary Fig. 7**a-c** were created by plotting cells with an out-of-plane magnetisation component $m_{\mathrm{z}} < 0$. The bottom images are z-slices through the centre of the simulated sample. The black and white plots in Supplementary Fig. 7**d-f** are averages of the out-of-plane magnetisation component m_{z} across the thickness of the sample, as shown in Fig. 3 of the main text.

The averaging process used to create the simulated images is most clearly visualised from Supplementary Fig. 8, where magnetisation profiles of a y-slice through the thickness of the simulated sample are shown. The top image of Supplementary Fig. 8 illustrates the in-plane y-component of the magnetisation together with isocontour lines. The $\theta = 90°$ curve refers to the $m_{\mathrm{y}} = 0$ surface, which encloses a skyrmion tube or a configuration pinned at the top surface, as shown at the top left of the image. The bottom plot of Supplementary Fig. 8 shows the average m_{z} component across the thickness of the sample (z-direction), $\langle m_{\mathrm{z}} \rangle$, where the strongest contrast is the positive out-of-plane component.

Zero values of the $\langle m_{\mathrm{z}} \rangle$ were computed using a linear interpolation of the curve and a root finding algorithm and are displayed as the dashed vertical lines. It can be seen that these zeros coincide with both the skyrmion tube centres and the middle point between two skyrmion tubes. Accordingly, the skyrmion centre zeros can be used to compute the inter-skyrmion distance and the distance of a skyrmion from the edge of the sample. In the main text the skyrmion-skyrmion and skyrmion-edge distances were both calculated using the zero at the centre of the rightmost skyrmion tube. In the case of skyrmion-skyrmion distance, it was computed from the zero of the

adjacent skyrmion at the left.

Supplementary Note 7: Three skyrmion tube states

To generate the simulated three skyrmion tube states presented in the main text, a function was applied to generate three paraboloid-shaped Bloch skyrmion precursors, with the core oriented opposite to the magnetisation of the rest of the field polarised sample. By defining

$$\mathbf{m} = (\sin\Theta\cos\Psi, \sin\Theta\sin\Psi, -\cos\Theta), \tag{1}$$

the functions $\Theta = \Theta(r, z)$, $\Psi = \Psi(\varphi)$, with (r, φ, z) as cylindrical coordinates, are specified as

$$\begin{aligned} \Theta(r, z) &= \frac{\pi}{r_{\text{par}}(z)} r, \quad r_{\text{par}}(z) = \sqrt{\frac{r_{\text{tube}}}{z_{\text{tube}}} z}, \quad 0 \leq z \leq 600\,\text{nm} \\ \Psi(\varphi) &= \varphi + \frac{\pi}{2}. \end{aligned} \tag{2}$$

Based on these functions, each of the three tubes was initialised with $r_{\text{tube}} = 40\,\text{nm}$ and $z_{\text{tube}} = 600\,\text{nm}$. The largest radius of the tube was specified with a value smaller than the helical length of FeGe due to the limited thickness of the 100 nm sample. The tube length was chosen according to the length of the tubes observed from the experimental images. To generate three tubes, the first tube was located with its centre at a distance of 125 nm from the edge of the sample and the second and third tubes were positioned with a 160 nm separation. The rest of the sample was initialised in a field-polarised state with magnetisation $\mathbf{m} = (0, +1, 0)$.

An overview of the initial state for the three tube system is shown in Fig. S9**a**. The regions within the $m_{\text{y}} = 0$ isosurfaces are shown in the top image and a z-slice at the centre of the sample is shown at the middle image, together with a simulated image at the bottom. This initial state

was relaxed at a range of magnetic fields by minimising the energy using a conjugate gradient algorithm implemented in the OOMMF code[9], and the resulting states are are shown in Fig. S9**b**-**f**. The 150 mT simulated state most resembles the three tube configuration seen in the experiments, displayed in Fig. S9**c**. Therefore, we chose this state to simulate the field sweep and observe the evolution of the skyrmion tubes with increasing and decreasing magnetic fields for comparison with the experimental data.

Supplementary Note 8: Randomly initialised field sweeps

In order to examine the possibility of skyrmion tube formation from a field sweep starting at 0 mT, we initialised a helical state at 0 mT from a fully randomised precursor state, performing an energy relaxation using the conjugate gradient minimisation method. The resulting initial state is shown in Fig. S10**a**, with m_y isosurfaces in the left plot and the average out of plane magnetisation through the thickness, $\langle m_\mathrm{z} \rangle$, in the right plot. Two field sweep processes were simulated starting from this initial zero field helical state, applying both an in-plane and an out-of-plane magnetic field. Results of the in-plane field sweep are shown in Fig. S10**a**-**e**. Images at the left side of these figures show the region within the $m_\mathrm{y} = 0$ isosurface. As the magnetic field is increased skyrmion tubes form with their ends pinned at the sample surfaces, which may be energetically favoured in comparison to the formation of Bloch points. Results of the out-of-plane field sweep are shown in Fig. S11**a**-**e**, with m_z isosurfaces depicted in the images to the left side of every field sweep stage. In this case, skyrmions are again primarily stabilised close to the boundaries of the sample, highlighting the importance of confinement and edge effects.

Supplementary Note 9: Details of the skyrmion tube and Bloch point state

A three dimensional representation of the simulated three skyrmion tubes state at 150 mT is shown in Fig. S12**a**, highlighting two cross sections through the width of the tubes, one near the edge of the simulated sample, and one at the location of the magnetic Bloch points. In Fig. S12**b** and **c**, cross sections close to the surface of the tubes at 280 and 150 mT are shown. White lines specify isocontours of the simulated magnetisation with a constant angle θ, which is measured with respect to the $m_\mathrm{y} = 1$ value, *i.e.* $\cos\theta = m_\mathrm{y}$. Isocontour surfaces have been used previously to show the asymmetric character of isolated skyrmion tubes embedded within a conical background, and highlight the Lennard-Jones like attraction when two adjacent tubes form a coupled state with overlapping isocontours[10,11], reaching an equilibrium distance. At higher applied magnetic fields, the background becomes field polarised, and the skyrmion tube isocontours no longer distort, causing two adjacent skyrmions to repel[11]. In the case of the confined skyrmion tubes analysed here, the observed structures are more complex as they end in a Bloch point singularity. Within the conical phase, besides the skyrmion tubes being slightly distorted along their lengths, there is no evidence of asymmetric isocontours surfaces, possibly due to the constrained sample dimensions. Despite this, the repulsion between neighbouring skyrmions and the sample edge with increasing magnetic field is still observed, in agreement with past calculations.

A profile of the topological charge density q of the surface cross section at 150 mT, is shown in Fig. S12**d**. The density of topological charge, or skyrmion number, is defined as

$$q = \frac{1}{4\pi}\mathbf{m}\cdot\left(\frac{\partial\mathbf{m}}{\partial z}\times\frac{\partial\mathbf{m}}{\partial x}\right). \tag{3}$$

In the cross section close to the surface of the skyrmion tube, shown by Fig. S12**d**, the topological charge density is given by the non-colinearity of the spins around the $\theta = 90^\circ$ region, and this is repeated through the skyrmion length. Towards the end of the skyrmion tubes, at the location of the magnetic Bloch points, the $\theta = 90^\circ$ area is reduced and q is concentrated in a infinitesimal region. Interestingly, just before and after the end of each skyrmion tube, the polarity of the charge density reverses from negative to positive, as shown by Figs. S12**e** and **f** respectively, revealing the singular character of the magnetic Bloch point. The simulated system is discretised with cuboid cells of 4 nm side length, hence it is likely that these Bloch point regions are not well defined and the energy cost of forming such a state may be underestimated. A better definition of this magnetisation requires an atomistic description, which is nontrivial due to the large size of the simulated sample.

Supplementary Note 10: Distortion of the skyrmion tube magnetisation profile

To characterise the distortion of the magnetisation profiles of the skyrmion tubes, we use the mathematics of the distortion of the helical state into a chiral soliton lattice with applied field[12,13]. We can write,

$$\frac{\mathrm{E}(\kappa)}{\kappa} = \sqrt{\frac{1}{h}}, \tag{4}$$

where $\mathrm{E}(\kappa)$ is the complete elliptical integral of the second kind, h is the ratio between the applied field and the critical field (H/H_C), and κ is the modulus of the elliptical function, for which we want to solve. Using the value of κ determined by minimising this equation, the magnetisation

profile of the distorted helical state for the vector $\vec{Q} \parallel \hat{x}$ and $\vec{H} \parallel \hat{z}$ is,

$$\begin{aligned} \mathbf{M} &= M_{\mathrm{s}}(0, M_{\mathrm{y}}, M_{\mathrm{z}}), \\ M_{\mathrm{y}} &= \sin(2\mathrm{am}(\sqrt{H}\frac{x}{\kappa}, \kappa)), \\ M_{\mathrm{z}} &= \cos(2\mathrm{am}(\sqrt{H}\frac{x}{\kappa}, \kappa)), \end{aligned} \tag{5}$$

where $\mathrm{am}(u, v)$ is the Jacobi amplitude function, and M_{s} is the saturation magnetisation. This can be conveniently expressed using the Jacobi Elliptic functions $\mathrm{sn}(u, v) = \sin(\mathrm{am}(u, v))$ and $\mathrm{cn}(u, v) = \cos(\mathrm{am}(u, v))$ as,

$$\begin{aligned} M_{\mathrm{y}} &= 2\mathrm{cn}(\sqrt{H}\frac{x}{\kappa}, \kappa)\mathrm{sn}(\sqrt{H}\frac{x}{\kappa}, \kappa), \\ M_{\mathrm{z}} &= \mathrm{cn}^2(\sqrt{H}\frac{x}{\kappa}, \kappa) - \mathrm{sn}^2(\sqrt{H}\frac{x}{\kappa}, \kappa). \end{aligned} \tag{6}$$

For the magnetisation profiles the relevant component of the magnetisation is M_{y}. This expression is then used to fit the magnetisation profiles, using the effective applied field h as a fitting parameter, and adding in an adjustable phase offset ϕ such that $x \to x + \phi$. This yields,

$$M_{\mathrm{y}} = 2\mathrm{cn}(\sqrt{H}\frac{x+\phi}{\kappa}, \kappa)\mathrm{sn}(\sqrt{H}\frac{x+\phi}{\kappa}, \kappa). \tag{7}$$

For the high field curves above 165 mT, this equation fits the simulated magnetization profiles. For the lower field curves, the distortion is seen to have the opposite directionality. This can be mathematically described by adding a negative sign and a phase offset of π, giving,

$$M_{\mathrm{y}} = -2\mathrm{cn}(\sqrt{H}\frac{x+\phi-\pi}{\kappa},\kappa)\mathrm{sn}(\sqrt{H}\frac{x+\phi-\pi}{\kappa},\kappa). \tag{8}$$

As shown in Fig. S13, the out-of-plane component of the magnetisation across the width of the SkT exhibits an asymmetrical distortion that varies as a function of applied magnetic field and is not observed in the conical state. The ratio of the applied field to the critical field, h, parameterises the magnitude of this distortion. We define $\nu = h$ for fields of 165 mT and above, and $\nu = -h$ for fields less than 165 mT, where the SkT state is essentially not distorted. This can be seen in Fig. S13**a**, where ν, obtained by fitting the magnetisation profiles with the chiral soliton lattice equations, is plotted as a function of applied magnetic field. The profiles of the SkT state reveal that the direction of the asymmetry is opposite at low and high magnetic fields. This is most easily visualised in Fig. S13**b** and **c**. At low applied fields, such as at 100 mT in Fig. S13**b**, the magnetisation profile varies fastest between the skyrmion tubes, due to the reduced separation of the tubes relative to their radii. Conversely, at high fields, such as at 270 mT in Fig. S13**c**, the magnetisation profile changes fastest within each skyrmion tube, due to the increased separation of the tubes relative to their radii. This leads to the magnetisation profile of the tubes appearing distorted, due to the varying skyrmion tube separation.

Supplementary References

1. Richardson, M. The partial equilibrium diagram of of the FeGe system in the range 40-72 at. % Ge, and the crystallisation of some iron germanides by chemical transport reactions. *Acta*

Chemica Scandinavica **21,** 2305–2317 (1967).

2. Bauer, A. & Pfleiderer, C. Magnetic phase diagram of MnSi inferred from magnetization and ac susceptibility. *Phys. Rev. B* **85,** 214418 (2012).

3. Popescu, H. *et al*. COMET: a new end-station at SOLEIL for coherent magnetic scattering in transmission. *J. Synchrotron Radiat.* **26,** 280-290 (2019).

4. Duckworth, T. A. *et al*. Magnetic imaging by x-ray holography using extended references. *Optics Express* **19,** 16223-16228 (2011).

5. Follath, R., Schmidt, J. S., Weigand, M. & Fauth, K. The Xray microscopy beamline UE46PGM2 at BESSY. *AIP Conference Proceedings* **1234,** 323 (2010).

6. Nolle, D. *et al*. Note: Unique characterization possibilities in the ultra high vacuum scanning transmission x-ray microscope (UHV-STXM) MAXYMUS using a rotatable permanent magnetic field up to 0.22 T. *Rev. Sci. Instrum.* **83,** 046112 (2012).

7. Blume, M. and Gibbs, D. Polarization dependence of magnetic x-ray scattering. *Phys. Rev. B* **37,** 1779 (1988).

8. Beleggia, M., Schofield, M. A., Volkov, V. V. & Zhu, Y. On the transport of intensity technique for phase retrieval. *Ultramicroscopy* **102,** 37–49 (2004).

9. Donahue, M. J. & Porter, D. G., OOMMF Users Guide, Version 1.0. *National Institute of Standards and Technology*, Gaithersburg, MD (1999).

10. Leonov, A. O., Monchesky, T. L., Loudon, J. C. & Bogdanov, A. N. Three-dimensional chiral skyrmions with attractive interparticle interactions. *J. Phys.: Condens. Matter* **28,** 35LT01 (2016).

11. Du, H. *et al*. Interaction of Individual Skyrmions in a Nanostructured Cubic Chiral Magnet. *Phys. Rev. Lett.* **120,** 197203 (2018).

12. Dzyaloshinskiĭ, I. E. Theory of Helicoidal Structures in Antiferromagnets. III. *Soviet Physics JETP* **20,** 665 (1965).

13. Izyumov, Y. A. Modulated, or long-periodic, magnetic structures of crystals. *Sov. Phys. Usp.* **27,** 845 (1984).
